# Cytoprotective and Neurotrophic Effects of Octadecaneuropeptide (ODN) in *in vitro* and *in vivo* Models of Neurodegenerative Diseases

**DOI:** 10.3389/fendo.2020.566026

**Published:** 2020-11-04

**Authors:** Olfa Masmoudi-Kouki, Amira Namsi, Yosra Hamdi, Seyma Bahdoudi, Ikram Ghouili, Julien Chuquet, Jérôme Leprince, Benjamin Lefranc, Taoufik Ghrairi, Marie-Christine Tonon, Gérard Lizard, David Vaudry

**Affiliations:** ^1^Laboratory of Neurophysiology Cellular Physiopathology and Biomolecule Valorisation, LR18ES03, Faculty of Sciences of Tunis, University Tunis El Manar, Tunis, Tunisia; ^2^Team Bio-PeroxIL, Biochemistry of the Peroxisome, Inflammation and Lipid Metabolism/University Bourgogne Franche-Comté (UBFC)/Inserm, Dijon, France; ^3^Normandy University, Neuronal and Neuroendocrine Differentiation and Communication, Inserm U1239, Rouen, France; ^4^Normandy University, Regional Platform for Cell Imaging of Normandy (PRIMACEN), Institute for Research and Innovation in Biomedicine (IRIB), Rouen, France

**Keywords:** gliopeptide ODN, neurodegeneration, cell protection, cell differentiation, oxidative stress

## Abstract

Octadecaneuropeptide (ODN) and its precursor diazepam-binding inhibitor (DBI) are peptides belonging to the family of endozepines. Endozepines are exclusively produced by astroglial cells in the central nervous system of mammals, and their release is regulated by stress signals and neuroactive compounds. There is now compelling evidence that the gliopeptide ODN protects cultured neurons and astrocytes from apoptotic cell death induced by various neurotoxic agents. *In vivo*, ODN causes a very strong neuroprotective action against neuronal degeneration in a mouse model of Parkinson's disease. The neuroprotective activity of ODN is based on its capacity to reduce inflammation, apoptosis, and oxidative stress. The protective effects of ODN are mediated through its metabotropic receptor. This receptor activates a transduction cascade of second messengers to stimulate protein kinase A (PKA), protein kinase C (PKC), and mitogen-activated protein kinase (MAPK)-extracellular signal-regulated kinase (ERK) signaling pathways, which in turn inhibits the expression of proapoptotic factor Bax and the mitochondrial apoptotic pathway. In N2a cells, ODN also promotes survival and stimulates neurite outgrowth. During the ODN-induced neuronal differentiation process, numerous mitochondria and peroxisomes are identified in the neurites and an increase in the amount of cholesterol and fatty acids is observed. The antiapoptotic and neurotrophic properties of ODN, including its antioxidant, antiapoptotic, and pro-differentiating effects, suggest that this gliopeptide and some of its selective and stable derivatives may have therapeutic value for the treatment of some neurodegenerative diseases.

## Introduction

The existence of binding sites for benzodiazepines (BZs), the most widely prescribed and therapeutically used drugs for their anxiolytic, sedative, and muscle relaxant properties, has prompted several teams to search for endogenous ligands of the BZ receptors. Thus, the team of Erminio Costa has isolated from rat brain extracts an 11-kDa polypeptide able to competitively displace tritiated diazepam on synaptosomes, which has been called diazepam binding inhibitor (DBI) (1). Two major compounds, generated from DBI, have been identified in rat brains, the triakontatetraneuropeptide (TTN, DBI_17−50_) and the octadecaneuropeptide (ODN, DBI_33−50_). DBI and its processing products, collectively grouped under the generic term of endozepines (2), are currently considered as the endogenous ligands of BZ receptors. DBI is also known as an acyl-coenzyme A-binding protein (ACBP) due to its ability to bind acyl-coenzyme A esters and to stimulate fatty acid synthesis in different cell types (3).

In this review, which will only refer to endozepines as natural ligands of BZ receptors, the acronym DBI will be used. In addition, we will focus on ODN, which is the major form of endozepines produced in the brain and whose structure has been well-preserved during evolution, suggesting that it exerts important biological functions in the central nervous system (CNS). Supporting this hypothesis, high concentrations of DBI mRNA and DBI-derived peptides are detected in the rat brain during ontogenesis. Furthermore, it has been reported that in the cerebellar cortex, ODN is expressed by Bergmann glia, which controls cerebellar granule neuron migration (4), indicating that ODN may act as a neurotrophic factor during brain development.

## Organization and Regulation of the Octadecaneuropeptide Gene Precursor (Diazepam Binding Inhibitor) Expression

Cloning and characterization of cDNAs encoding DBI were first performed from a rat brain cDNA library (5). Since then, DBI has been cloned from other tissues (adrenal, liver, and testis) and from various animal species, including human (6), cattle (7), frog (8), and carp (9). The cDNA encoding DBI has also been cloned in invertebrate species such as the fruit fly *Drosophila melanogaster* (10), the butterfly *Bombyx mori* (11), and the tobacco maggot *Manduca sexta* (12), different plant species such as *Arabidopsis thaliana* (13), and unicellular organisms such as the yeast *Saccharomyces cerevisiae* (14) and the bacteria *Escherichia coli* (15, 16).

**Figure 1:**
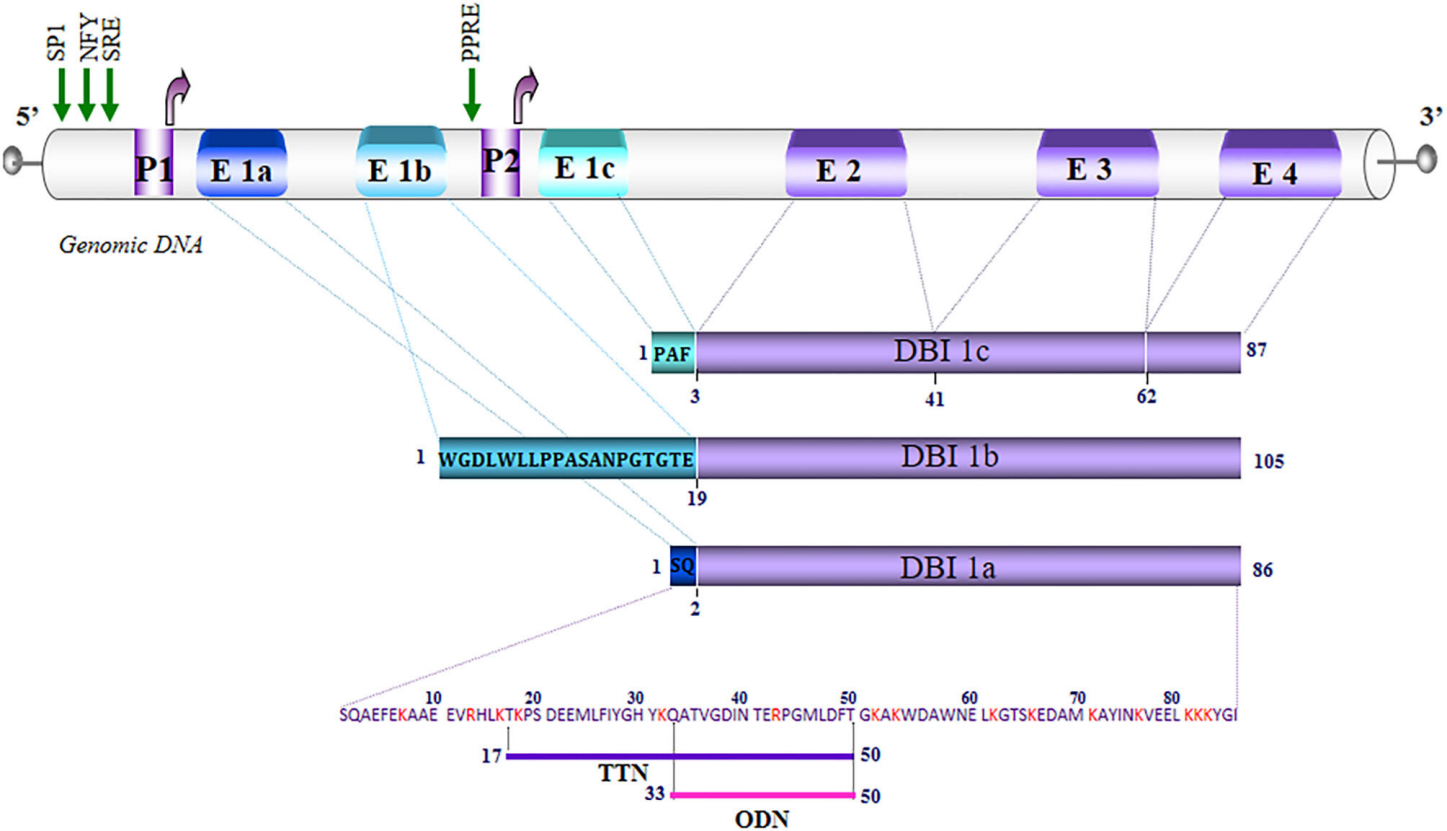
Schematic representation of the organization of the diazepam-binding inhibitor (DBI) gene in humans. The exons (E) are numbered from 1 to 4. Alternative splicing of exon 1, which is composed of three sub-exons E1a, E1b, E1c, generates DBI 1a (86 amino acids), 1b (105 amino acids), and 1c (87 amino acids). The letters in the blue rectangles correspond to the amino acid sequence of the regions translated by E1a, E1b, and E1c. The P1 promoter region controls the expression of DBI 1a and 1b. The P1 and P2 promoters control the expression of DBI 1c. The green arrows correspond to the sites of transcriptional initiation from promoters P1 and P2. The regions that are common to the three DBI isoforms are represented by rectangles in purple. The basic residues are noted in red, and the underlined amino acids correspond to the sequences of triakontatetraneuropeptide (TTN) (DBI_17−50_) and octadecaneuropeptide (ODN) (DBI_33−50_). NFY, nuclear transcription factor Y; SP1, transcription factor specificity protein 1; SRE, sterol response element; PPRE, peroxisome proliferator-response element.

Southern blot analysis of genomic DNA from human (6), rat (5), and mouse (17) revealed the existence of several genes encoding DBI, but only one of which is functional. In human, the active gene is located on the q12-21 region of the long arm of chromosome 2 (18) and the pseudogenes are located on chromosomes 5, 6, 11, and 14 (19). The human and murine genes are organized into four exons (E1–E4), coding for regions 1–2, 3–41, 42–62, and 63–86 of the DBI, respectively (20, 21), while in *Drosophila*, there are only three exons (10). In human and murine adipocytes, a novel exon, named E1c, has been identified in the DBI gene, which codes with the three other exons for a longer transcript. Genomic DNA analysis shows that the E1c exon is located in the intron between exons E1 and E2, and that its expression would be under the control of a second promoter located in intron 1 (22, 23). These data reinforce the work of Helledie et al. (24), which showed that the DBI gene, cloned from human and murine adipocytes, contains a functional consensus sequence on intron 1, the peroxisome proliferator-activated receptor (PPAR)-response element (PPRE), capable of binding the transcription factors PPARγ and retinoid X receptor α. All these data indicate that the expression of the DBI gene is under the control of two promoter regions (P1 and P2) located upstream and downstream of the E1 exon initially described in human and rat (Figure 1).

The absence of TATA and CCAAT boxes, the presence of several element initiators of transcription, and the high content of nucleotide acids C and G in a promoter are often considered as the hallmarks of a domestic gene. Nevertheless, the expression level of DBI gene is regulated in different cell types (25). In addition, the P1 and P2 regions of the DBI gene have several consensus sequences for transcription factors including a glucocorticoid-response element (GRE), a CAAT-binding transcription factor/nuclear factor 1 (CTF/NF1), a hepatocyte nuclear factor (HNF), a sterol regulatory element-like sequence (SRE), and a PPAR sequence. Moreover, it has been reported that glucose and hormones, such as insulin or androgens, regulate the expression of the DBI gene (21, 26–28). Several research groups have also shown a modification of the levels of DBI gene expression in the brain in various experimental conditions. For instance, a decrease in DBI mRNA levels is observed after starvation (29) or castration (30) in rat, while an increase of DBI expression is shown after high-frequency vestibular stimulation in rabbits (31). Drug addiction (alcohol, morphine, and nicotine) also leads to an increase in DBI gene expression in the rodent brain (32, 33), and abrupt interruption of the consumption of these substances further increases DBI mRNA levels (33). *In vitro* studies on cultured astrocytes have shown that DBI gene expression is stimulated by different cell stress inducers such as peptide β-amyloid (34) and hydrogen peroxide (H_2_O_2_) (35). All these observations reveal that the DBI gene cannot be considered as a “housekeeping gene.”

## Distribution of Octadecaneuropeptide Family Peptides in the Nervous System

### At Tissue Level

In all vertebrate species studied, endozepines are present both in the CNS and in many peripheral organs and tissues (25, 36). Although DBI and its derivatives are found in the gonads (126 pM), duodenum (100 pM), kidneys (73 pM), heart (30 pM), liver (22 pM), skeletal muscle (18 pM), adrenals (15 pM), lungs (13 pM), and spleen (11 pM) (25), the highest concentrations are found in rat brain (10–50 μM). Peptides of the ODN family are widely distributed in the brain, with the highest levels measured in the olfactory bulb, hypothalamus, hippocampus, cerebellum, striatum, cerebral cortex, and circumventricular organs (Table 1).

**Table 1:** Octadecaneuropeptide-like immunoreactivity (ODN-LI) content in different rat brain structures.

**Brain region**	**ODN-LI level (ng/region)**
Olfactory bulb	57.2
Cerebral cortex	427.8
Hippocampus	55.7
Striatum	28.5
Cerebellum	258.6
Hypothalamus	73.6

### At the Cellular Level

A limited number of immunocytochemical studies using antibodies against DBI show a localization of endozepines in neurons (37, 38). However, the majority of the data in the literature indicate that endozepines are primarily expressed in glial cells (25). For example, ODN- and/or DBI-like immunoreactivities are detected in astrocytes in many brain areas including the cerebral cortex, hippocampus, amygdala, and olfactory bulb (39, 40), in the ependymocytes bordering the cerebral ventricles (39, 41), in the tanycytes of the median eminence (39, 42), in Bergmann cells from the cerebellum (40), and in Gomori-positive astrocytes from the arcuate nucleus (43). Similarly, in the peripheral nervous system, immunostaining for ODN and/or DBI is associated with Schwann cells (44). Finally, in the retina, endozepines are exclusively expressed by specialized glial cells, the Müller cells (45).

## Regulation of Octadecaneuropeptide Release

Ultrastructural studies revealed that DBI immunoreactivity is diffused throughout the cytoplasm (39), excluding its packaging in secretory vesicles. This is consistent with the absence of signal peptide in the primary sequence of DBI that could direct the protein into the endoplasmic reticulum/Golgi system. Nevertheless, mass spectrometry analysis of rat astroglial cell secretome revealed the presence of DBI in incubation media (46). The endozepine secretion is insensitive to brefeldin A, an inhibitor of Golgi vesicular transport (46), and can be stimulated by induction of autophagy by rapamycin (47). These data indicate that DBI and its derived peptides may be released by a mechanism independent of the conventional vesicular exocytosis pathway. The translocation of endozepines to the extracellular space could be provided by carriers called ATP-binding cassettes (ABCs), as demonstrated for the release of interleukin (IL)-1β by astrocytes (48) and annexin 1 by follicle-stellar cells (49), two proteins whose precursors are devoid of a signal peptide sequence. Consistent with this hypothesis, a site-directed mutagenesis study conducted with the amoeba *Dictyostelium discoideum* has shown the existence of a direct interaction between DBI and ABC-serine protease transporter (TagA) (50).

**Figure 2:**
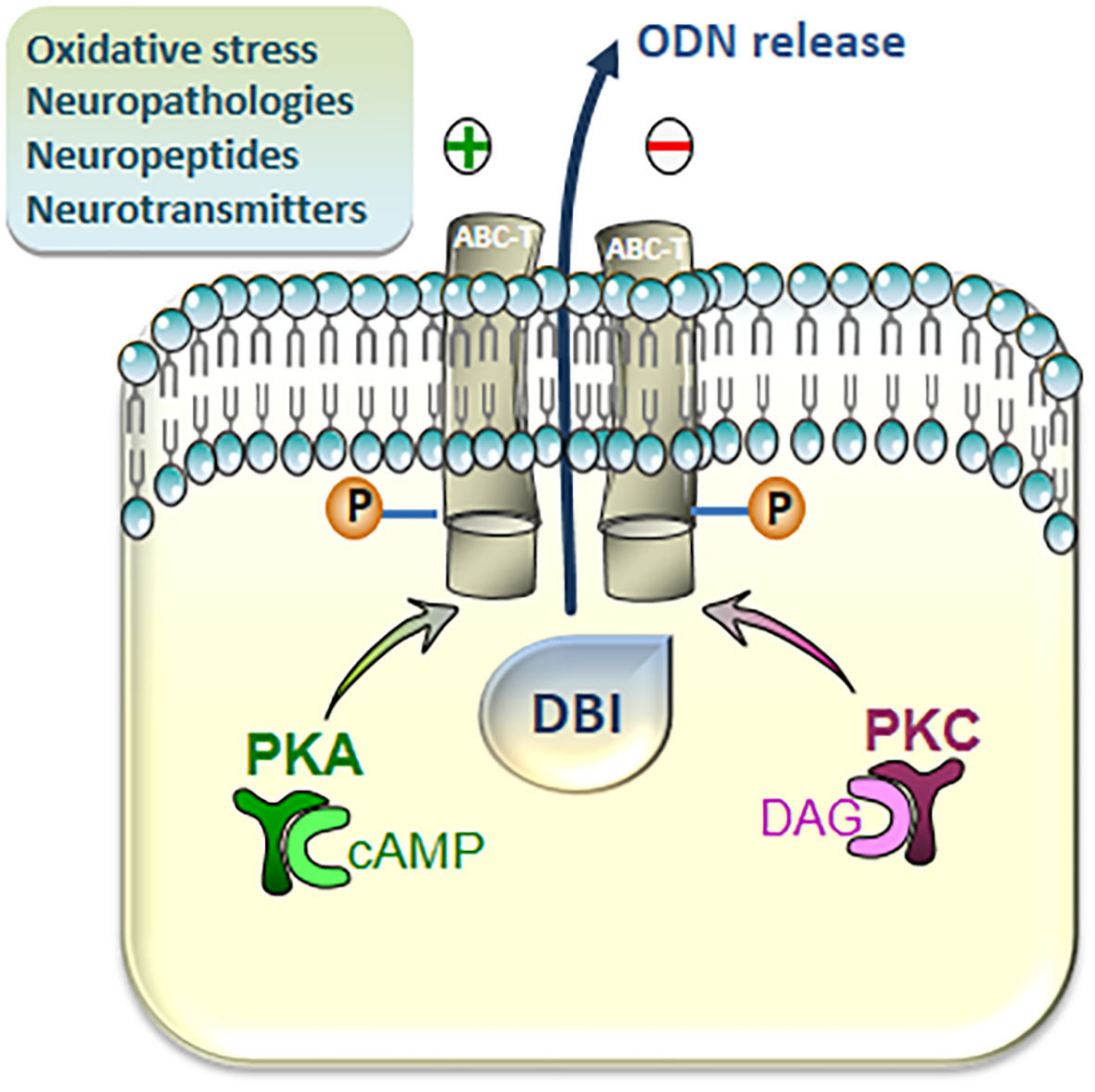
Intracellular pathways involved in the regulation of the release of diazepam-binding inhibitor (DBI)-related peptides from cultured astroglial cells. Activation of cAMP/protein kinase A (PKA) and Diacylglycerol (DAG)/protein kinase C (PKC) cascades stimulates the release of DBI-related peptides through phosphorylation, at consensus sites, of transmembrane ATP-binding cassette transporters (ABC-Ts). The production of the octadecaneuropeptide (ODN), which occurs extracellularly, is finely regulated (positively or negatively) by various neuronal mediators, including classical neurotransmitters and neuropeptides, and 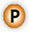 under cerebral pathologies and oxidative injuries.

Although the mechanism of secretion of ODN and other DBI-derived peptides is not yet clearly elucidated, the secretion of endozepines is modulated in different pathophysiological situations and regulated by many factors. An assay using antibodies against ODN shows that the level of ODN-like immunoreactivity (ODN-LI) is increased in the plasma of septic shock patients (51). *In vitro* studies reveal that the release of ODN-LI is positively regulated by numerous neuroactive compounds, including neuropeptides [pituitary adenylate cyclase-activating polypeptide (PACAP) (52), urotensin-II (UII), UII-related peptide (53), and peptide β-amyloid (54)], steroid hormones [cortisol, pregnenolone, or progesterone (55)], cytokines [tumor necrosis factor-α and IL-1β (56)], or elevated extracellular potassium concentrations (57), and conversely repressed by the neuropeptide somatostatin (58) and the neurotransmitter gamma aminobutyric acid (GABA) (59). Pharmacological studies indicate that the release of ODN-LI from astroglial and retinal cells is mediated by ABC transporter activity through a mechanism that is dependent on the phosphorylation by protein kinases A and C (PKA and PKC) (52, 54, 60) (Figure 2), and blockage of the phosphorylation of ABC transporter inhibits the basal release of ODN-LI by about 50%. A phosphorylated form of ODN is also released by astrocytes but appears less active than the non-phosphorylated peptide, suggesting the existence of a posttranslational regulation mechanism (61).

In culture media from astrocytes exposed to moderate oxidative stress, the quantity of authentic ODN is significantly higher than that detected in culture media from control astrocytes (35). Clinical studies have shown that the levels of endozepines, ODN, and DBI, increase in the plasma or cerebrospinal fluid (CSF) of patients suffering from pathological disorders related to oxidative stress, such as systemic inflammation, hepatic encephalopathy, and neurodegenerative diseases (25). Such an increase in ODN release could be responsive for the stimulation of antioxidative defenses (induction of Mn-superoxide dismutases, catalase, glutathione peroxidase-1, sulfiredoxin-1 gene transcription, and glutathione biosynthesis) in glia and neurons (62, 63) in agreement with the emerging concept indicating that ODN acts as a potential cytoprotective actor preventing the deleterious action of oxidative insults.

## Biological Activities of Octadecaneuropeptide in the Central Nervous System

As endogenous ligands of BZ receptors, ODN interacts with the central-type BZ receptors, which is an integrated component of the GABA-A receptor (GABA-A-R)/Cl^−^ channel complex (64, 65). Like DBI, ODN has been shown to displace diazepam and modulate GABAergic transmission *via* an allosteric reaction (1). Electrophysiology and cell functional studies indicate that ODN may act as an agonist, i.e., a positive allosteric modulator (PAM) promoting the action of GABA, or as an inverse agonist, i.e., a negative allosteric modulator (NAM) reducing the action of GABA, depending on the subunit composition of the GABA-A-R (57, 66). Studies conducted on cultured astrocytes have demonstrated that ODN is also the endogenous ligand of a G protein-coupled receptor, which is functionally and pharmacologically different from the conventional BZ receptors (67, 68). In particular, ODN stimulates the metabolism of polyphosphoinositides (PIPs) in rat astrocytes and cerebellar granule neurons *via* a phospholipase C (PLC) coupled to a G protein sensitive to pertussis toxin (63, 69). In addition, in cultured astrocytes, ODN increases the intracellular concentration of calcium from intracellular pools (68, 70). In the same cell type, ODN stimulates the formation of cAMP through activation of adenylyl cyclase (AC) activity (61, 71). The ability of ODN to stimulate phosphorylation of extracellular signal-regulated kinase 1 and 2 (ERK 1 and 2) is also found in both astrocytes and cerebellar granule neurons (63, 71). These data collectively indicate that ODN is the natural and specific ligand of a G_i/0_ or G_S_ protein-coupled receptor, which activates complementary transduction mechanisms depending on the cell type.

The structure–activity studies of ODN in relation to its ability to activate the metabotropic receptor expressed in rat astrocytes show that the C-terminal octapeptide of ODN (OP, ODN_11−18_) is the shortest biologically active fragment of the peptide (72). OP is able to mimic the effects of ODN on calcium mobilization and cAMP level increase observed in astrocytes (71, 72). Conversely, OP has no effect on the binding of [^3^H]flumazenil in cerebellar granular cells or of [^3^H]PK11195 in rat astrocytes (73). The effects of ODN on the activation of the cAMP/PKA, PIPs/calcium/PKC, and MAPK-ERK transduction pathways in astrocytes and neurons are completely suppressed by a specific antagonist of this receptor, the cyclo_(1−8)_[DLeu^5^]OP (63, 68, 72).

### Effect of Octadecaneuropeptide on Cell Proliferation

ODN, *via* activation of the central-type BZ receptors, stimulates the incorporation of [^3^H]thymidine into cultured rat astrocytes (74). Consistent with this proliferative effect of ODN, *in vivo* studies have demonstrated that ODN promotes the proliferation of neuronal progenitor stem cells from the germinative sub-ventricular zone in adult rat (75). Conversely, inhibition of DBI gene transcription by shRNA transfection *in vivo* reduces the number of proliferating cells and the number of neurons newly formed at the level of olfactory bulbs but does not affect stem cell survival (75). Furthermore, the fact that ODN can inhibit neuronal cell death and stimulate neurogenesis suggests that it could also play a key role during brain development. In support of this hypothesis, it has been shown that, in the cerebellar cortex, endozepines are exclusively expressed by Bergmann glia (2, 4), which control cerebellar granule neuron migration. Taken together, these data indicate that ODN may exert a neurotrophic effect and reinforce the notion that ODN may promote cell proliferation, survival, and/or differentiation.

### Protective Effects of Octadecaneuropeptide on Neuronal Cells

ODN has been shown to rescue neurons and glial cells from neurotoxicity induced by several substances such as H_2_O_2_ (35, 71, 76, 77), 6-hydroxydopamine (6-OHDA) (62, 63), and 1-methyl-4-phenyl-1,2,3,6-tetrahydropyridine (MPTP) (35). Indeed, exposure of cultured granule neurons and N2a neuronal cell line to very low concentrations (in the subpicomolar range) of ODN totally abolishes the deleterious effects of 6-OHDA (63) and H_2_O_2_ (76– 78). ODN also exerts a strong protective effect against oxidative stress-induced apoptosis on cultured astrocytes (62, 78, 79). ODN acts by preventing (i) the accumulation and overproduction of intracellular reactive oxygen species (ROS), (ii) the depletion of glutathione (GSH) levels, and (iii) the decrease of the expression and activity of the antioxidant enzymes provoked by oxidative stress (79). Furthermore, ODN prevents apoptotic cell death by inhibiting (i) the overexpression of the proapoptotic protein Bax as well as the repression of the antiapoptotic protein Bcl-2 and (ii) the drop of the mitochondrial membrane potential responsible for the stimulation of caspase-3 activity. Treatment of neuroblastoma cell lines and cerebellar granule neurons with astrocyte-conditioned medium significantly promotes neuron survival under oxidative injury induced by 6-OHDA (80) and H_2_O_2_ (76, 81). Treatment of cerebellar granule neurons with ODN metabotropic receptor antagonist greatly attenuated the protective action of astrocyte-conditioned medium. Quantitative measurement of ODN by mass spectrometry indicates that the amount of ODN present in glial conditioned medium is in the same range of concentration as the one necessary for the neuroprotective action of the peptide on granular neurons against apoptotic cell death induced by oxidative damage, suggesting a neuroprotective effect of the endogenous gliopeptide (76).

Some behavioral studies have shown that in the same way as ODN, intracerebroventricular administration of TTN induces proconflict- and anxiety-related behavior. At the cellular level, TTN also promotes proliferation and intracellular calcium increase in glial cells (70, 74). However, the potential neuroprotective and/or neurotrophic activity of TTN has never been reported.

The neuroprotective activity of ODN has also been observed *in vivo*, in an MPTP mouse model of Parkinson's disease (35). A single intracerebroventricular injection of 10 ng ODN, 1 h after the last administration of MPTP, is sufficient to prevent the loss of dopaminergic neurons in the substantia nigra pars compacta (SNpc) induced by the toxin and to block the degeneration of nerve fibers in the striatum (35). ODN-mediated neuroprotection is associated with a reduction of the number of glial fibrillary acidic protein-positive reactive astrocytes and a strong inhibition of the expression of pro-inflammatory genes induced by MPTP in the SNpc. Moreover, ODN blocks the inhibition of the antiapoptotic gene Bcl-2 and the stimulation of the proapoptotic genes Bax and caspase-3 (35). ODN also prevents the accumulation of ROS and lipid oxidation products both in the SNpc and the striatum. Furthermore, DBI^−/−^ mice exhibit more vulnerability to MPTP injection than wild-type animals (DBI^+/+^). Thus, ODN-knockout (KO) mice are more sensitive to MPTP-induced inflammatory and oxidative brain damages, suggesting that the endogenous ODN may also be neuroprotective (35). These data indicate that the induction of ODN production in pathological conditions, similar to the one observed at the early stages of neurodegenerative processes, may correspond to a compensatory mechanism, initiated by reactive astrocytes, to reduce their sensitivity to oxidative aggression and to limit the progression of brain damages.

Altogether, these results demonstrate that, based on its antioxidative (77, 82), anti-inflammatory (35), and antiapoptotic effects (63, 71), the gliopeptide ODN, which acts as a potent neuroprotective agent, could lead to the development of effective therapeutic agents for the treatment of cerebral injuries involving oxidative neurodegeneration. It should nevertheless be pointed out that in the acute phase of stroke, ODN, through its allosteric modulation of GABA A receptor, boosts the excitability of cortical neurons and as a consequence increases neuronal damages (83). Consistent with this observation, DBI^−/−^ mice exhibit increased infarct volume compared to wild-type animals. However, when administered in the subacute period after stroke, ODN improves functional recovery, showing that if provided at the right time, ODN can also be of interest for the treatment of stroke patients.

### Differentiating Activity of Octadecaneuropeptide on N2a Cells

**Figure 3:**
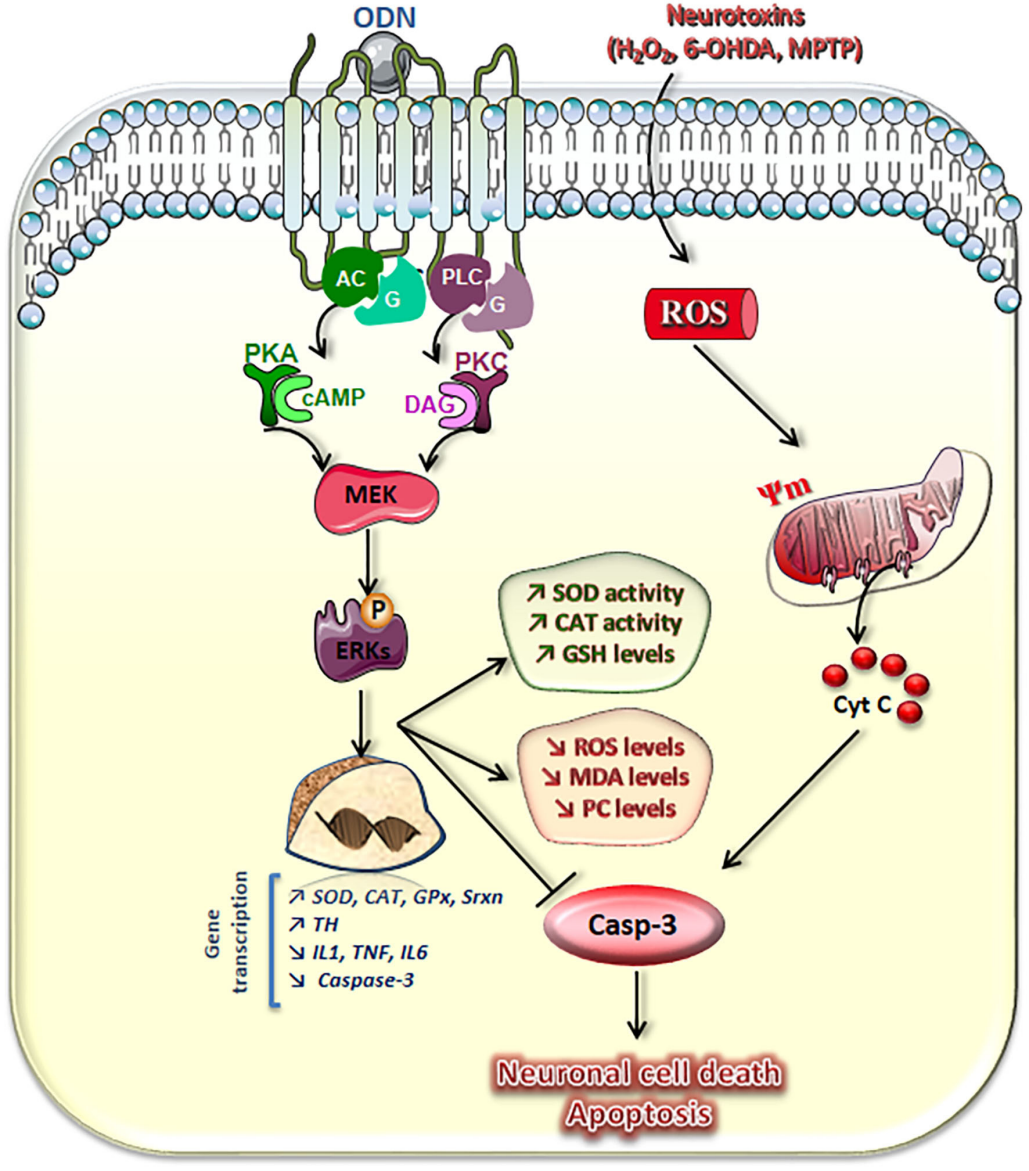
Schematic representation of transduction pathways involved in the neuroprotective effects of octadecaneuropeptide (ODN). ODN, through activation of its metabotropic receptor and the AC–protein kinase A (PKA), phospholipase C (PLC)–protein kinase C (PKC), and mitogen-activated protein (MAP)-kinases/extracellular signal-regulated kinase (ERK) signaling pathways, stimulates superoxide dismutase (SOD), and catalase (CAT) activities and glutathione (GSH) cellular contents, which prevent the drop of the mitochondrial membrane potential (Ψ) and activation of caspase-3 (Casp-3) induced by hydrogen peroxide (H_2_O_2_) or 6-hydroxydopamine (6-OHDA) in cultured neurons and astrocytes. ODN also abolished neurotoxin-induced overproduction of reactive oxygen species (ROS) and blocked oxidative damage of cell molecules, i.e., formation and accumulation of lipid oxidation products [malondialdehydes (MDAs)] and protein carbonyl compounds (PCs). Concomitantly, in cultured astrocytes, ODN stimulates *Mn-SOD, CAT, glutathione peroxidase-1* (GPx), and *sulfiredoxin-1* (Srxn) gene transcription and rescues 6-OHDA-associated reduced expression of endogenous antioxidant enzymes. In 1-methyl-4-phenyl-1,2,3,6-tetrahydropyridine (MPTP)-treated mice, ODN prevents the degeneration of dopaminergic neurons in the *substantia nigra pars compacta*, abrogates the effect of toxin on inhibition of *tyrosine hydoxylase* (TH) gene transcription, and blocks the stimulation of the expression of pro-inflammatory genes, such as *interleukins (IL) 1* and *6, tumor necrosis factor*-α *(TNF-*α*)*, and the proapoptotic gene *caspase*-*3*. Taken together, these results show that the gliopeptide ODN exerts a potent neuroprotective effect through mechanisms inhibiting oxidative stress, neuroinflammation, and apoptosis.

There is evidence indicating that neuropeptides, such as PACAP (84, 85), brain-derived neurotrophic factor (BDNF), and nerve growth factor (NGF) (86), promote both neuronal survival and differentiation, suggesting that the gliopeptide ODN, which protects neurons and astrocytes from apoptosis (25, 80), could also have a differentiating activity. We have thus tested whether ODN can have a neurotrophic property on murine N2a cells and stimulate neurite (axons and dendrites) outgrowth, in indded, at very low concentrations (in the fentomolar range, 10^−14^ M), ODN exerts a protective action on H_2_O_2_-induced N2a cell apoptosis and induces cell differentiation in the presence or absence of fetal bovine serum (87) (Figure 3). The cytoprotective effects of ODN on N2a cells (capacity to prevent damages induced by oxidative stress) are observed at similar concentrations to those inducing neuronal differentiation (35, 63), which leads us to propose that ODN can be considered as a potent neurotrophic factor. It is noteworthy that no cytotoxic effect (inhibition of cell growth and cell adhesion, loss of transmembrane mitochondria potential, ROS overproduction, loss of lysosomal integrity, or cell death induction) is observed with ODN in the concentration range inducing neurite outgrowth (87). During neuronal differentiation, cells also acquire excitability and express genes with functional identity (88).

To define the signaling pathway associated with the differentiating effect of ODN on N2a cells, inhibitors that are linked to the pathways involved in the cytoprotective action of ODN, such as the PKA inhibitor H89, the PLC inhibitor U73122, the PKC inhibitor chelerythrine, and the MEK inhibitor U0126, were used. All these molecules were able to block ODN differentiating activity (79, 87), indicating that ODN's cytoprotective and differentiating activities use common signaling pathways. The MEK–ERK pathway may be activated by both PKA and PKC (63, 71). Some other pathways such as the phosphoinositide 3-kinase (PI3K)/Akt involved in cell differentiation and activated by other neurotrophic factors could also contribute to ODN-induced N2a cell differentiation.

**Figure 4:**
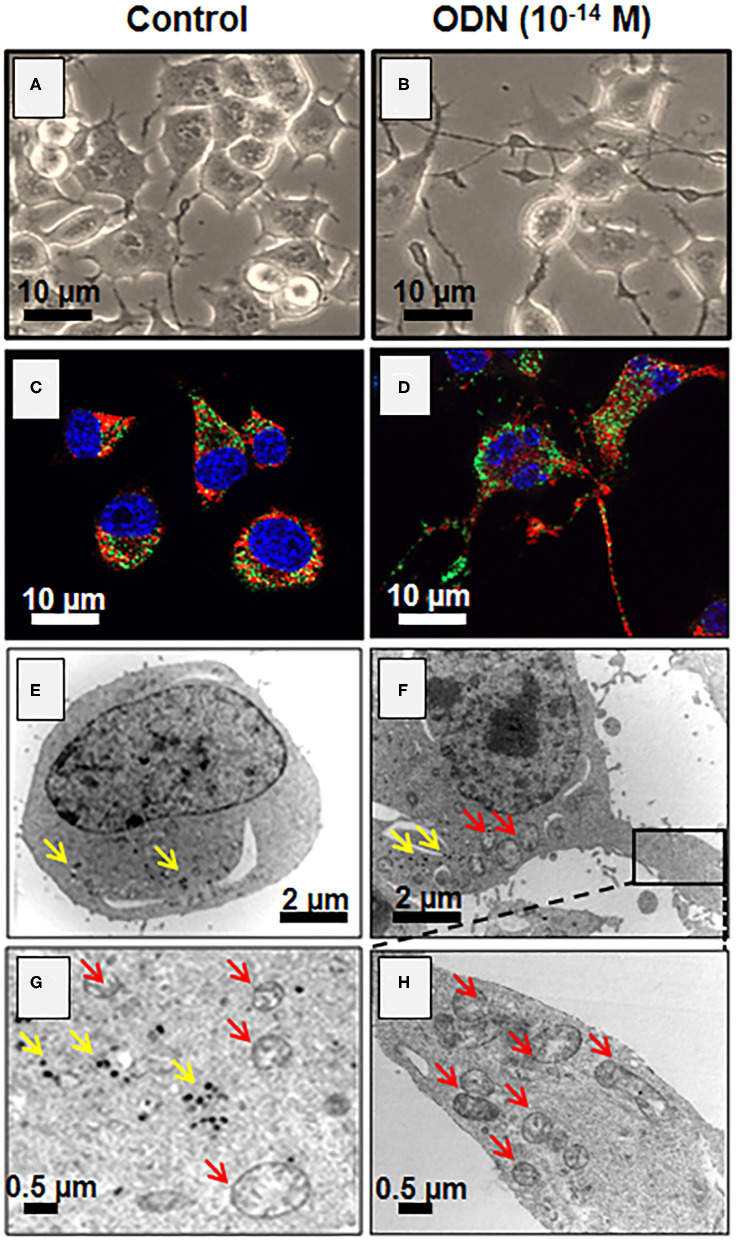
Induction of neuronal differentiation in octadecaneuropeptide (ODN)-treated N2a cells. Murine neuronal N2a cells were treated with ODN (at 10^−14^ M for 48 h). **(A,B)** Undifferentiated [control, **(A)**] and differentiated cells (characterized by morphological aspects based on neurite outgrowth, **(B)** cells were observed by phase contrast microscopy. Due to the presence of numerous mitochondria and peroxisomes in neurites, these later can be considered functional. **(C,D)** The mitochondria were detected with Mitotracker Red (red fluorescence) and the peroxisomes by indirect immunofluorescence after staining with an antibody raised against the ABCD3 peroxisomal transporter (green fluorescence) in control **(C)** and ODN-treated cells **(D)**. The nuclei were counterstained with Hoechst 33342; the images were acquired under a fluorescent microscope coupled with an Apotome (Zeiss). The green spots point toward peroxisomes, and red spots point toward mitochondria**. (E–H)** Visualization by transmission electron microscopy of mitochondria (red arrows) and peroxisomes (yellow arrows) in control **(E,G)** and differentiated N2a cells [treated with ODN, **(F,H)**].

Since the growth of neurites (dendrites and/or neurons) requires significant energy and lipid synthesis, the effect of ODN on mitochondria and peroxisomes was investigated (89–91). In the presence of ODN, topographical changes, especially regarding mitochondria distribution in the N2a cells, are observed not only in the soma but also in neurites (dendrites and/or axons) (Figure 4). It is well-established that mitochondria are capable of producing fatty acids and cholesterol, which are essential for neurite outgrowth, and that peroxisome also contributes to cholesterol biogenesis *via* the production of acetyl-CoA (92, 93). Noteworthy, higher levels of cholesterol and its precursors (lanosterol, desmosterol, and lathosterol) are detected in cells treated with ODN (10^−14^ M) (87). It should be noted that there is a preference for the Bloch pathway for the cholesterol biogenesis as shown by the increased levels of lathosterol and desmosterol after treatment with ODN (87). Altogether, these data provide some of the mechanisms involved in the differentiation of the neuronal cell line N2a by ODN.

## Transduction Pathways Involved in the Neuroprotective Effects of Octadecaneuropeptide

Pharmacological studies have shown that central BZ receptor agonists are unable to mimic the protective effects of ODN (25). Similarly, central-type BZ receptors and translocator protein (TSPO) antagonists fail to suppress ODN-induced protection of neurons and glial cells from injuries, indicating that “classical” BZ receptors are not involved in the protective activity of ODN. However, OP and cyclo_(1−8)_OP, two agonists of the ODN metabotropic receptor, exhibit a protective activity when used in the same range of concentration as ODN. In addition, cyclo_(1−8)_[DLeu^5^]OP, a potent antagonist of this metabotropic receptor, blocks the protective effects of ODN on neurons and astrocytes (62, 63, 71, 82).

Downstream of the receptor, several transduction pathways can mediate the protective effect of ODN according to the cell type. In astrocytes, activation of ODN metabotropic receptor implies stimulation of the activity of the AC/cAMP/PKA pathway (71). The neuroprotective effect of ODN against 6-OHDA-induced cerebellar granule cell death involves activation of the PLC/IP3/PKC pathway (63). Downstream to PKA and PKC activations, ODN stimulates the phosphorylation of the ERK1 and ERK2 proteins in astrocytes and neurons (79). The phosphorylation of ERK1 and ERK2 in turn stimulates the expression of the Bcl-2 protein and concomitantly inhibits the expression of Bax, thereby preventing mitochondrial dysfunction and blocking cell death *via* the intrinsic apoptotic pathway (84, 94). The stimulating effect of ODN on the expression of antioxidant enzyme genes and their enzymatic activities is dependent on the activity of the cAMP/PKA transduction pathway as well as that of MAPK–ERKs in astrocytes (62, 77, 82). ODN promotes cerebellar granule cell survival from apoptosis through the PKC pathway (63), while another neuropeptide, PACAP, protects the same neurons from apoptosis through the PKA pathway (95). It would thus now be of interest to investigate if combining the two molecules leads to an enhanced and/or prolonged antiapoptotic effect.

## Octadecaneuropeptide, An Endogenous Neuroprotective Agent

Clinical studies have revealed that endozepine levels are significantly increased in the CSF of patients suffering from neurological disorders such as epilepsy, hepatic encephalopathy (96, 97), or Alzheimer's and Parkinson's diseases (98). Mass spectrometry, radioimmunoassay (RIA), and q-RT PCR analyses reveal that moderate oxidative stress induces stimulation of the endogenous production of ODN, as well as the expression of the ODN precursor gene DBI from cultured astrocytes (76). Furthermore, induction of endogenous ODN production is responsible for (i) the stimulation of the expression and activity of antioxidant enzymes and thus rapid resorption of ROS, (ii) the protection of bio-macromolecules from oxidative damages, and (iii) the prevention of astrocytes and neuron cell death induced by oxidative stress. In contrast, inhibition of ODN release by the PKA inhibitor H89 or blockage of the effects of ODN with an antagonist of its metabotropic receptor, the cyclo_(1−8)_[DLeu^5^]OP, exacerbates damages induced by oxidative stress on astroglial cell viability (76). Consistently, knockdown of ODN precursor expression, by DBI siRNA, induces morphological alterations with loss of membrane integrity and the formation of apoptotic bodies and increases the vulnerability of oxidative stress inducing cell death (76). It has also been shown, by using DBI-KO animals, that ODN precursor deficiency increases brain sensitivity to MPTP toxicity, highlighting the neuroprotective role of endogenous ODN against neuronal degeneration (35). Taken as a whole, these data indicate that the induction of ODN production in pathological conditions reproducing early stages of neurodegenerative processes may represent a compensatory mechanism, initiated by reactive astrocytes, to reduce their sensitivity, as well as that of their surrounding neurons, to oxidative aggression and to limit the progression of the neurodegeneration process in some neurological disorders.

## Author Contributions

OM-K, AN, GL, JL, M-CT, and DV provided valuable editorial comments and relevant bibliographic references. YH, SB, IG, and DV provided valuable editorial comments. OM-K wrote the manuscript with the contribution of AN, GL, and DV. TG, JC, and BL contributed to the review and editing revised version. All authors contributed to the article and approved the submitted version.

## Conflict of Interest

The authors declare that the research was conducted in the absence of any commercial or financial relationships that could be construed as a potential conflict of interest.
